# Synthesis, Antibacterial, and Anti HepG2 Cell Line Human Hepatocyte Carcinoma Activity of Some New Potentially Benzimidazole-5-(Aryldiazenyl)Thiazole Derivatives

**DOI:** 10.3390/molecules23123285

**Published:** 2018-12-11

**Authors:** Mohamed E. Khalifa, Adil A. Gobouri, Fahad M. Kabli, Tarik A. Altalhi, Abdulraheem S. A. Almalki, Mahmoud A. Mohamed

**Affiliations:** 1 Department of Chemistry, faculty of Science, Taif University, Taif 21974, Saudi Arabia; agobouri00@yahoo.com (A.A.G.); fmk_37@hotmail.com (F.M.K.); tmmba@windowslive.com (T.A.A.); drasaalmalki1@gmail.com (A.S.A.A.); 2 Department of Biochemistry, Faculty of Agriculture, Cairo University, Giza 12613, Egypt; elcamel.mahmoud@gmail.com

**Keywords:** benzimidazoles, antimicrobial activity, antitumor, HepG2 cell line, pathogenic bacteria

## Abstract

The paper describes the synthesis and biological evaluation of some new benzimidazole derivatives as potent clinical drugs that are useful in the treatment of some microbial infections and tumor inhibition. The starting compound 2-(bromomethyl)-1*H*-benzimidazole (**1**) was prepared, and hence underwent interesting functionalization reactions to afford several series of benzimidazole-5-(aryldiazenyl)thiazole derivatives: **3a**–**c**, **7a**–**c**, and **8a**–**c**. The antibacterial activities of the synthesized compounds were evaluated by calculation of the inhibition zone diameter (mm) and the determination of minimum inhibitory concentration (µg/mL) against selected pathogenic bacteria *Staphylococcus aureus* (Gram-positive bacteria) and *Escherichia coli* (Gram-negative bacteria).Noticeable efficiency was found based on in vitro screening for their antioxidant activity and cytotoxicity effect against the human liver cancer cell line (HepG2) and human hepatocyte carcinoma cells at relatively high concentrations.

## 1. Introduction

The promising therapeutic potential of benzimidazole derivatives could be traced back to 1944and Woolley, who speculated that benzimidazole could act similar to purines to elicit some biological responses [1]. The optimization of substituents around the benzimidazole nucleus resulted in many drugs with useful therapeutic activities such asmethyl (5-benzoyl-1*H*-benzimidazol-2-yl)carbamate (mebendazole) as parasitic worm infestations, (*RS*)-2-([3-methyl-4-(2,2,2-trifluoroethoxy)pyridin-2-yl]methylsulfinyl)-1*H*-benzo[d]imidazole (lansoprazole) and (*RS*)-6-(difluoromethoxy)-2-[(3,4-dimethoxypyridin-2-yl)methylsulfinyl]-1*H*-benzo[d]imidazole (pantoprazole) as proton pump inhibitors; 1-[(4-fluorophenyl)methyl]-*N*-[1-[2-(4-methoxyphenyl)ethyl]-4-piperidyl]benzoimidazol-2-amine (astemizole) as antihistaminic[2]; triazole-thiazol derivatives as analgesic [3,4,5,6]; 2-[2-(4-chlorophenyl)-1,3-benzoxazol-5-yl]propanoic acid (benoxaprofen) as anti-inflammatory [4,5,6,7]; methyl [5-(propylthio)-1*H*-benzoimidazol-2-yl]carbamate (albendazole) as antimicrobials [8,9,10]; 1-(isopropylsulfonyl)-6-(1-phenylprop-1-en-1-yl)-1*H*-benzo[d]imidazol-2-amine (enviradine) as antiviral [11,12]; antitubercular [13,14]; 4-(1*H*-1,3-benzodiazol-2-yl)-1,3-thiazole(thiabendazole) as antihelmenthic [15,16,17,18]; picoline-imidazoles as anticonvulsant [19,20];4-[5-[bis(2-chloroethyl)amino]-1-methylbenzimidazol-2-yl]butanoic acid (bendamustine) as anticancer [21,22,23]; 5-methoxy-2-[(4-methoxy-3,5-dimethylpyridin-2-yl)methanesulfinyl]-1*H*-benzimidazole (omeprazole) as antiulcer [24,25]; angiotension-II receptor antagonists [26]; 2-ethoxy-1-([4-[2-(2*H*-1,2,3,4-tetrazol-5-yl)phenyl]phenyl]methyl)-1*H*-1,3-benzodiazole-7-carboxylic acid (candesarten) as antihypertensive [27]; alkynyl benzimidazoles as modulators of metabotropic glutamate receptors [3,16,17,18,24,28,29,30]; and *N*-ribosyl-dimethyl benzimidazole as an axial ligand for cobalt in vitamins [19,31,32].Aiming to prepare heterocyclic systems with some role in biological applications, we report in the present work the synthesis of novel derivatives of benzimidazoles by convenient methods. In vitro screening for their antimicrobial activities was determined against selected pathogenic bacteria, namely *Staphylococcus aureus* (Gram-positive bacteria) and *Escherichia coli* (Gram-negative bacteria) using inhibition zone diameter (mm) and minimum inhibitory concentration methodologies comparing with *Kanamycin* and *Ampicillin*, respectively, as standard antibacterial agents. Their antioxidant and antitumor activities against the HepG2 human hepatocyte carcinoma cell line were also evaluated.

## 2. Results

Benzimidazoles are hybrid heterocyclic compounds that are formed from fused benzene and imidazole rings with broad industrial, biological, and clinical applications. The associated rich literature with benzimidazole moieties prompted us to synthesize new functionalized benzimidazole derivatives and study their potential as bioactive compounds, continuing our previous efforts related to the synthesis of new pharmacological compounds [33,34,35,36,37,38,39,40,41].

### 2.1. Chemistry

**Scheme 1:**
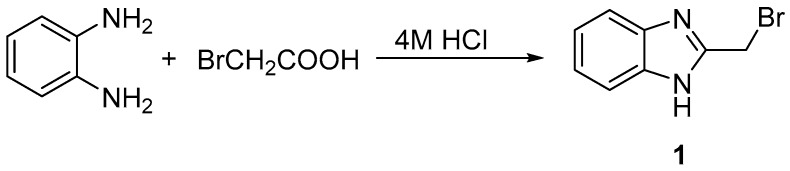
Synthesis of 2-(bromomethyl)-1*H*-benzimidazole (**1**).

Synthesis of the starting 2-(bromomethyl)-1*H*-benzimidazole (**1**) proceeded smoothly according to the reported method by the reaction of *o*-phenylenediamine with bromoacetic acid in the presence of 4 M hydrochloric acid, as shown in Scheme 1.

The IR spectrum of 2-(bromomethyl)-1*H*-benzimidazole (**1**) revealed intense IR bands at 3372–3025 cm^−1^ (NH, =CH aromatic, and –CH aliphatic). The ^1^H-NMR spectrum displayed a singlet signal at *δ* 4.85 ppm due to the methylene protons (2H, CH_2_–Br), a singlet at *δ* 10.32 ppm due to the NH–benzimidazole proton, and at *δ* 7.21–7.60 ppm due to the aromatic protons. The spectral analysis was found to be in good agreement with the literature [42].

**Scheme 2:**
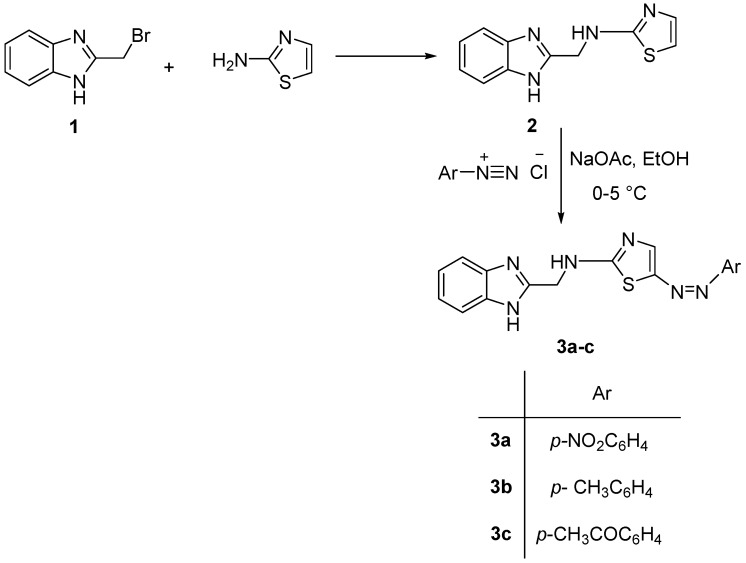
Synthesis steps for the preparation of benzimidazole derivatives(**3a**–**c**).

The direct condensation of 2-(bromomethyl)-1*H*-benzimidazole (**1**) with 2-aminothiazole was carried out to give the *N*-((1*H*-benzimidazol-2-yl)methyl)thiazol-2-amine (**2**) [43],which underwent diazo coupling with different aryl diazonium chorides, yielding the corresponding *N*-((1*H*-benzimidazol-2-yl)methyl)-5-(aryldiazenyl)thiazol-2-amines (**3a**–**c**), as shown in Scheme 2.

The structures of the isolated compounds (**2** and **3a**–**c**) were secured by their spectral analysis as described in the experimental section, where a characteristic singlet signal at *δ* 6.90 ppm due to the C_4_-thiazole proton was displayed.

**Scheme 3:**
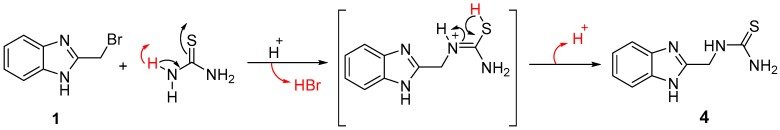
Mechanism of formation of alkylthiourea benzimidazole derivative (**4**).

The reaction of thiourea with α-haloalkyl or α-halocarbonyl compounds often proceed through the sulfur atom due to its high nucleophilicity to give thiouronium salts [44]. Whereas, conducting the reaction under acidic medium (e.g., trifluoroacetic acid) afforded the alkylthiourea compounds [45,46,47]. Thus, the reaction of 2-(bromomethyl)-1*H*-benzimidazole (**1**) with thiourea in acidic medium gave the previously reported derivative 1-((1*H*-benzimidazol-2-yl)methyl)thiourea (**4**) [45] through the suggested mechanism in Scheme 3. The structure of the obtained compound was secured by its spectral analysis, and was in good agreement with the literature.

**Scheme 4:**
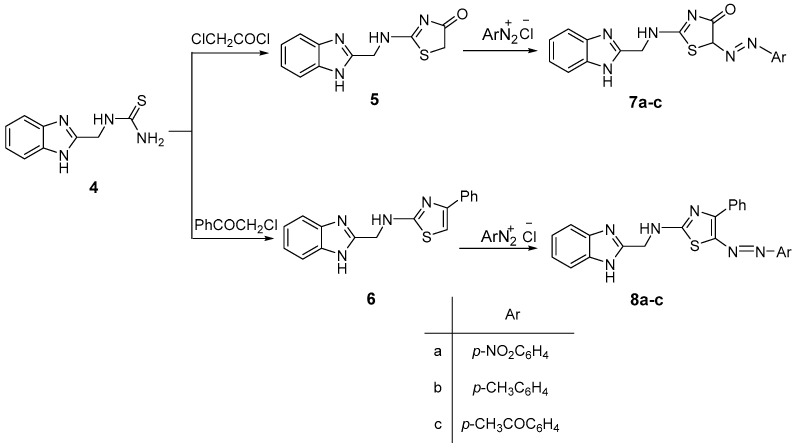
Synthesis of arylazo derivatives (**7a**–**c**) and (**8a**–**c**). The formation of compounds (**7a**–**c** and **8a**–**c**) was elucidated by their spectral analyses.

The thiourea derivative **4** was then incorporated in a set of investigations aiming at exploiting the reactivity of the thioureido moiety to build up the target compounds. Thus, 1-((1*H*-benzimidazol-2-yl)methyl)thiourea (**4**) was cyclized into the thiazole derivatives 2-(((1*H*-benzimidazol-2-yl)methyl)amino)thiazol-4(5*H*)-one (**5**) and *N*-((1*H*-benzimidazol-2-yl)methyl)-4-phenylthiazol-2-amine (**6**), by treating with α-halocarbonyl compounds (i.e., chloroacetyl chloride and phenacyl chloride). The aminothiazolyl derivatives (**5** and **6**) were then allowed to couple with different aromatic diazonium chloride compounds, affording the target arylazo compounds; 2-(((1*H*-benzimidazol-2-yl)methyl)imino)-5-(4-aryl)diazenyl)thiazolidin-4(5*H*)-one derivatives (**7a**–**c**) and *N*-((1*H*-benzimidazol-2-yl)methyl)-5-(4-aryl)diazenyl)-4-phenylthiazol-2-imine derivatives (**8a**–**c**) respectively, as shown in Scheme 4.

### 2.2. Biological Impact of the Synthesized Compounds

#### 2.2.1. Antibacterial activity

**Figure 1:**
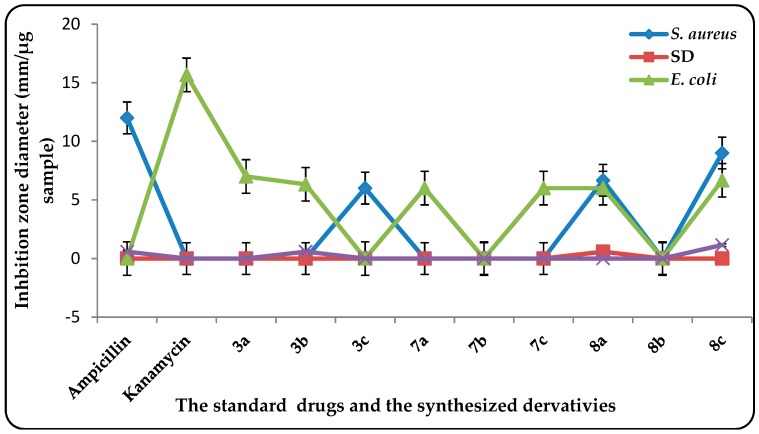
Antimicrobial activity of the tested benzimidazole derivatives vis-à-vis selected pathogenic bacteria strains comparing with standard drugs.

The antibacterial activities of novel synthesized compounds (100 µg/mL in DMSO), were determined in vitro by using the disc diffusion method [48] against *Staphylococcus aureus (S. aureus) ATCC 25923* as Gram-positive bacteria and *Escherichia coli (E. coli) ATCC 25922* as Gram-negative bacteria compared with the standard drugs *Kanamycin* and *Ampicillin*, respectively. The results of the assessments are plotted in Figure 1.

#### 2.2.2. Minimum Inhibitory Concentration (MIC)

The Minimum Inhibitory Concentration for the synthesized compounds was determined using a spot-on-lawn assay [49]. The collected data are listed in Table 1. The lowest MIC value is the highest antibacterial effect against the target bacterial strain.

The antibacterial study showed that some of the tested compounds exhibited variable antimicrobial activities against both bacteria Gram-negative and Gram-positive bacteria at a concentration of 100 µg/mL. The presence of substituted electron withdrawing groups such as nitro and acetyl groups in compounds **3c**, **8a**, and **8c** showed activities against *E. coli* (Gram-negative bacteria), while most of the synthesized compounds exhibited moderate activity against *S. aureus* (Gram-positive bacteria).

Compound **8c** showed the highest affect against tested bacterial strains compared with the rest of the synthesized benzimidazoles under consideration due to the presence of an electron-withdrawing group (COR group) on the phenyl ring. It should be noted that the thiazolone moiety resulted in a dramatic reduction of the antibacterial efficiency (e.g., compounds **7a**–**c**).

#### 2.2.3. Antioxidant Potential of the Synthesized Compounds

##### DPPH Radical Scavenging Assay

**Figure 2:**
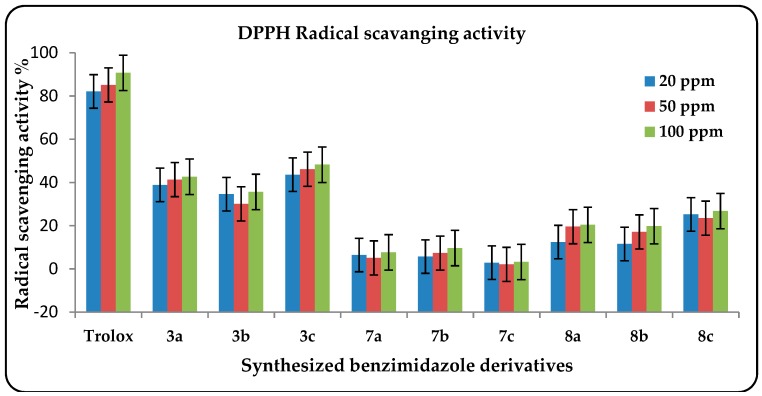
Inhibition percentage average results of DPPH radical by the tested synthesized benzimidazoles based on three independent experiments.

**Figure 3:**
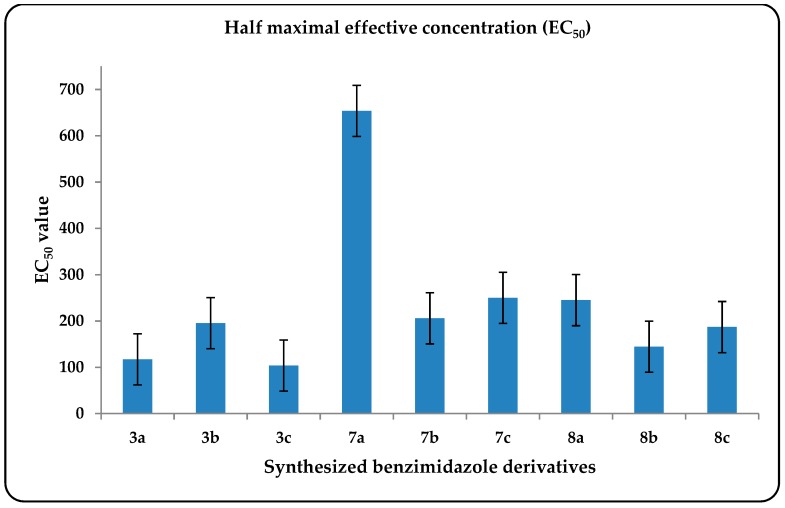
The average results of EC_50_ for the tested synthesized benzimidazoles based on three independent experiments.

Antioxidant capacities for the synthesized compounds were screened using a DPPH free radical scavenging assay incorporating a metastable free radical that is capable of accepting hydrogen radicals from antioxidants in solution. The method is based upon different mechanisms to provide complementary insight into the antioxidant activity of the synthesized compounds [50]. The reaction between DPPH and antioxidants can be monitored by the decrease in the absorbance of the violet-colored free radicals. Inhibition percentages of DPPH radical by selected tested compounds were plotted in Figure 2, and the measure of drug potency in terms of half maximal effective concentration (EC_50_) values were plotted in Figure 3 (i.e., the compound concentration where 50% of its maximal effect is observed). The high antioxidant and scavenging activities of the tested compounds may be due to the resonance delocalization of the nitrogen lone pairs through the double bonds. This structure may lead to radical formation at more than one site, especially on the benzene ring attached to the electron-withdrawing groups (e.g., **3a** and **3c**: the highest DPPH value and the lowest EC_50_ value, respectively). This conclusion is also supported by previously reported results [51].

#### 2.2.4. Evaluation of Cytotoxicity Effects of Benzimidazoles and its Derivatives Against the Human Liver Cancer Cell Line (HepG2).

**Figure 4:**
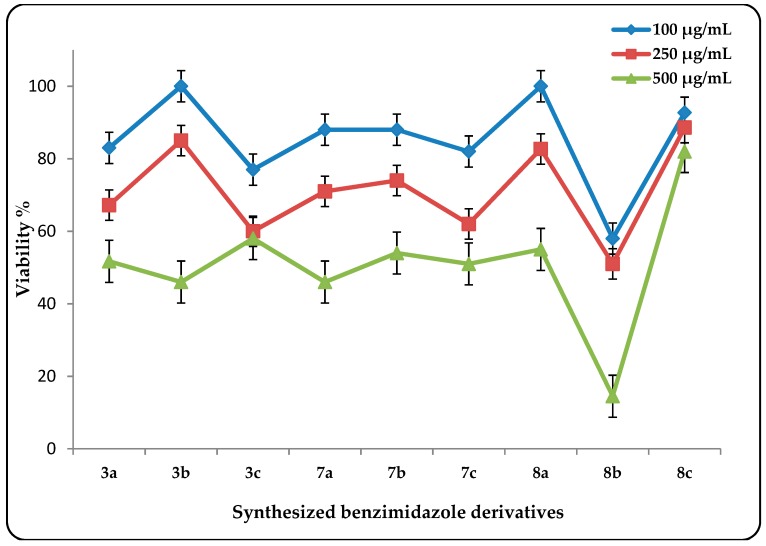
The viability % of human liver cancer (HepG2) cell line treated with the synthesized benzimidazoles at 100 μg/mL, 250 μg/mL, and 500 μg/mL based on three independent experiments.

All of the compounds were screened in vitro for possible potential antitumor molecules against human liver cancer cell line (HepG2) and showed variable anticancer activity. The Neutral Red (NR) technique [52] was used to evaluate the cytotoxic effects of gradient concentrations (100 μg/mL to 500 μg/mL) of the tested compounds on the human liver (HepG2) cell line and in terms of cell viability percentage, which measured the potency of the compound in inhibiting tumor growth (Figure 4).

As shown in Figure 4, the tested compounds exhibited antitumor activities in different concentrations, where increasing the concentration led to less percent viability. The benzimidazole derivatives (**3**) and (**8**) in common showed higher activity than the other selected compounds that were tested. The presence of the 4-phenyl function with or without attached electron-withdrawing groups increased the antitumor activity due to their high antioxidant and free radical scavenging ability (e.g., **3a** and **8b**). This also could be explained by the previously reported facts [51], and is supported by the literature [53].

## 3. Materials and Methods

### 3.1. Chemical Reagents

All of the chemical reagents that were used were of analytical grade or chemically pure and supplied by Sigma Aldrich Co., Darmstadt, Germany.

### 3.2. Micro and Spectroscopic Analysis

All of the corrected melting points are in degree centigrade and measured using a Stuart SMP20 melting point apparatus (Bibby Scientific Limited, Staffordshire, UK). The infrared spectra were recorded on a PerkinElmer Alpha platinum-ATR spectrometer; the ^1^H-NMR (300 MHz) and ^13^C-NMR (75 MHz) spectra were recorded on a Varian Mercury VXR-300 spectrometer (Varian Inc., Palo Alto, CA, USA) and the chemical shifts were related to that of the solvent DMSO-*d*_6_ using tetramethylsilane (TMS) as an internal standard. MS spectra (HRMS) were acquired on TRACE GC Ultra gas chromatograph mass spectrometry, coupled with a THERMO mass spectrometer detector ISQ Single Quadrupole Mass Spectrometer (THERMO Scientific Corp., Waltham, MA, USA)and were obtained by electron ionization (EI) at 70 eV, using a spectral range of *m*/*z* 50–1000.All of the microanalyses and spectral analyses were performed by the Micro Analytical Centers of Taif University-Saudi Arabia (IR spectra, HRMS), Cairo University (^1^H-NMR, ^13^C-NMR), and National Research Center-Egypt (Mass spectra).

### 3.3. Biological Analysis

All of the used kits for biochemical parameters were supplied by the Diagnostic Company, Maidenhead, UK. All of the chemicals that were used were supplied by Gibco^®^ Life Technologies, Waltham, MA, USA. The biological tests were performed by the biotechnology unit, faculty of Agriculture-Cairo University, Egypt. Human transformed cell lines from liver (HepG2) were obtained from the Egyptian Company for Vaccine and Serum (VACSERA, Cairo, Egypt). The statistical package for social science (SPSS) 1999 program version (IBM, Armonk, NY, USA) was used for all of the analyses [54].

#### 3.3.1. Antibacterial Evaluation

The disc diffusion procedure was used to evaluate the antibacterial activity of the synthesized compounds [53]. Uninoculated agar medium was poured in sterilized Petridishes and left for solidifying. One hundred milliliters of trypticase soy agar + 0.6% yeast extract were melted and cooled to 50 °C, and then inoculated by the culture of the examined microorganism. Inoculated medium was poured over the previous layer and left to solidify at 4 °C (surface layer should be constant in volume and horizontally homogenous). Discs of Whatman No. 1 filter paper (6.0 mm in diameter) were sterilized by autoclaving at 121 °C for 15 min. Each individual tested compound was dissolved in 10% dimethyl sulfoxide (DEMSO) with a final concentration 100 mg/mL. An accurate volume (15 μL) of each tested compound (100 mg/mL) was aseptically added to each disc and left to dry. Each disc was aseptically placed on the middle of an agar plate surface (in triplicate) and left at 4 °C. Fifteen μL of DEMSO (10%) was aseptically added to the disc and used as control. Then, 15 μL of *Kanamycin* (30 μg/mL) was aseptically added to the disc and used as a positive standard reference for Gram-positive strains, and *Ampicillin* (10 μg/mL) was used for Gram-negative strains. The inhibition clear zones (mm) were recorded in all of the samples and equal zero in no-inhibition results.

#### 3.3.2. Determination of Minimum Inhibitory Concentration (MIC)

The minimum inhibitory concentration (MIC) of synthesized compounds was assayed by using a spot-on-lawn assay [49]. Cells of laboratory strains (LAB strains) were grown in de Man, Rogosa & Sharpe MRS broth (Oxoid) supplemented with 0.5% CaCO_3_ at 30 °C with shaking (120 strokes per min (SPM)) to the early stationary phase, and the initial pH of the medium was adjusted to pH 6.0. Culture fluid was obtained by removing the cells by centrifugation, and was filtered with a cellulose acetate membrane filter (pore size 0.2 µm; Advantec, Tokyo, Japan). The resulting sample was serially diluted twofold with MRS broth (pH 5.0). Soft nutrient agar (20 mL) was solidified in a sterile dish (140 mm by 100 mm) after the addition of 60 µL of an overnight culture of *Listeria monocytogenes* SUB635. This strain was used as an indicator in the assay, since a clearer inhibitory zone was obtained with this organism than with *B. subtilis*. After 30 min of drying, 10 µL of each diluted sample was spotted onto the plate. The resulting plates were incubated at 30 °C overnight, and the titer was defined as the reciprocal of the highest dilution (2*^n^*) that resulted in the inhibition of the indicator lawn. Thus, the arbitrary unit (AU) of antibacterial activity per milliliter was defined as 2*^n^* × 1000 µL/10 µL.

To examine the spectrum of activity of the purified bacteriocin against various bacterial strains, the test described above was performed with soft agar containing cells of test strains grown in a suitable medium to the early stationary phase. The abilities to inhibit a certain bacterial strain were compared by using the highest dilution that resulted in a growth inhibition zone.

#### 3.3.3. Assay of Cytotoxicity

Neutral Red (NR) assay [55] was used to evaluate the cytotoxicity effects of prepared compounds. 30.000 Cells/well were plated in 96-well plates in quadruplets for each treatment. 24 h later, cells were washed with pre-warmed phosphate-buffered saline (PBS) and 125 μL of media with an appropriate concentration of each synthesized compound. Afterwards, cells were washed with pre-warmed PBS, and 125 μL of the NR reagent was added in each well. The plates were incubated at 37 °C with 5% CO_2_, for a further 5 h. After incubation, plates were taken out, gently washed three times with PBS, and 100 μL of NR desorb solution was added per well. Plates were protected from light and shaken on a shaker at 60 rpm for 30 min, followed by keeping the plates still for 5 min. Plates were taken to a 96-well plate reader, and their absorbance was measured in each well at 540 nm. Each value is based on means of the absorbance values acquired from the quadruplet of treatments and normalized to the mean value of untreated control and expressed as % cell death according to the following equation (Equation (1)):

(1) $$\%\;\mathrm{Cell}\;\mathrm{death}=\frac{{\mathrm{Abs}}_{540}\;\mathrm{treated}\;\mathrm{sample}}{{\mathrm{Abs}}_{540}\;\mathrm{untreated}\;\mathrm{sample}}\times 100$$

The data is based on three independent experiments.

#### 3.3.4. Antioxidant Potential

The tested compounds were analyzed for their antioxidant potential according to the reported technique [56]. The stock solution of 1,1-diphenyl-2-picrylhydrazyl (DPPH) was prepared by dissolving 24 mg of DPPH in 100 mL of methyl alcohol (MeOH), and then stored at 20 °C in the dark. The working solution was obtained by diluting 10 mL of stock solution with 45 mL of MeOH, in order to obtain an absorbance of 1.1 ± 0.1 units at 515 nm. A volume of 10 µL of different synthesized compounds’ concentrations (20 µg/mL, 50 µg/mL, and 100 µg/mL) was added to 990 µL of 0.094 mM DPPH freshly working solution up to total volume of one mL. Assays were continuously monitored at 515 nm over a one-hour period at 25 °C. Changes in absorbance were minimal for all of the samples after 50 min. The antioxidant abilities were expressed as µM Trolox equivalents. Each sample was analyzed in triplicate. The inhibition percentage of the DPPH radical by the samples was calculated according to Equation (2):

(2) $$\%\;\mathrm{Inhibition}=\frac{\left[\mathrm{Ab}-\mathrm{Aa}\right]}{\mathrm{Ab}}\times 100$$

where, Ab is the absorption of the blank sample (t = 0 min), and Aa is the absorption of the tested compound or standard substance solution (t = 30 min). The EC_50_ value is defined as the concentration of antioxidant in the reactive system that is necessary to decrease the initial DPPH concentration by 50%, and is calculated from the data obtained.

### 3.4. Synthesis and Structural Analysis

#### 3.4.1. General Procedure for the Synthesis of 2-(Bromomethyl)-1*H*-Benzimidazole (**1**)

The 2-bromomethyl benzimidazole (**1**) was prepared according to the literature procedure [48,57,58], where *O*-phenylenediamine (2.16 g, 20 mmol) was dissolved in 30–40 mL of four M of HCl by heating. The bromoacetic acid (4.16 g, 30 mmol) was added thoroughly, and then, the reaction mixture was boiled under reflux for five h. The mixture was neutralized with ammonia, and the solid product was filtered, washed with water, and dried. The obtained product was recrystallized from acetone to give red crystals. M.p.: 60–62 °C (59–61 °C [42]), yield: 3.6 g, 86%.

#### 3.4.2. Synthesis of *N*-((1*H*-Benzimidazol-2-yl)Methyl)Thiazol-2-Amine (**2**)

A mixture of 2-(bromomethyl)-1*H*-benzimidazole **1** (2.11 g, 10 mmol), 2-aminothiazole (1 g, 10 mmol), and potassium iodide KI (1.66 g, 10 mmol) in ethanol (50 mL) was heated under reflux for 6 h. Afterwards, an aqueous solution of potassium hydroxide KOH (1.66 g, 10 mmol, in five mL of H_2_O) was added and heating was continued for a further 2 h. The cold reaction mixture was poured onto ice-cold water, and the precipitated solid product was filtered off, dried, and recrystallized from ethanol to give reddish brown crystals of *N*-((1*H*-benzimidazol-2-yl)methyl)thiazol-2-amine (**2**). M.p.: > 250 °C (398 °C [43]), yield: 1.6 g, 69%.

#### 3.4.3. General Procedure for the Synthesis of the *N*-((1*H*-Benzimidazol-2-yl)Methyl)-5-(Aryldiazenyl)Thiazol-2-Amine (**3a**–**c**)

The corresponding aryl diazonium chlorides were prepared by adding cold sodium nitrite solution (0.69 g, 10 mmol in 10 mL of H_2_O) to a cold suspension of 10 mmol of different aromatic amines (namely *p*-nitroaniline, 1.38 g; *p*-toluidine, 1.07 g; and *p*-aminoacetophenone, 1.35 g) in concentrated HCl (4–6 mL) with stirring. To a cold solution of *N*-((1*H*-benzimidazol-2-yl)methyl)thiazol-2-amine **2** (2.3 g, 10 mmol) in ethanol (40 mL) and sodium acetate (1.6 g, 20 mmol), a cold aqueous solution from the corresponding aromatic diazonium salts was added dropwise with stirring at 0–5 °C for 2 h. The solid products obtained were filtered off, washed with water followed by cold ethanol, dried, and recrystallized from ethanol to give *N*-((1*H*-benzimidazol-2-yl)methyl)-5-(aryldiazenyl)thiazol-2-amine (**3a**–**c**).

*N-((1H-benzimidazol-2-yl)methyl)-5-((4-nitrophenyl)diazenyl)thiazol-2-amine* (**3a**). Brown solid, M.p.: >250 °C, Yield: 2.4 g, 63%. IR: *ν*_max_ = 3740–2850 (NH, =CH aromatic, –CH aliphatic), 1620 (C=N), 1451(CH_2_) and 1335, 1569 (NO_2_) cm^−1^. ^1^H-NMR: *δ*/ppm = 3.51 (s, 2H, CH_2_ aliphatic), 5.87 (s, 1H, NH), 6.95–7.65 (m, 8H, Ar–H; s, 1H, C_4_–thiazole–H), 10.90 (s, 1H, NH–benzimidazole ring). ^13^C-NMR: *δ*/ppm = 43.2, 115.2 (2C), 123.0 (2C), 123.8 (2C), 126.6 (2C), 134.7, 138.8 (2C), 140.7, 141.5, 147.2, 147.8, 163.2. MS (*m*/*z*, %): 379 (M^+^, 100.0). HRMS calc. for C_17_H_13_N_7_O_2_S: 379.0851, found: 379.0873.

*N-((1H-benzimidazol-2-yl)methyl)-5-(p-tolyldiazenyl)thiazol-2-amine* (**3b**). Brown solid, M.p.: >250 °C, Yield: 1.8 g, 54%. IR: *ν*_max_ = 3740–2850 (NH=CH aromatic, –CH aliphatic), 1620 (C=N), 1453 (CH_2_), and 1389 (CH_3_) cm^−1^. ^1^H-NMR: *δ*/ppm = 2.49 (s, 2H, CH_2_ aliphatic), 3.40 (s, 3H, CH_3_), 5.87 (s, 1H, NH), 6.92–7.64 (m, 8H, Ar–H; s, 1H, C_4_–thiazole–H), 10.99 (s, 1H, NH–benzimidazole ring). ^13^C-NMR: *δ*/ppm = 21.3, 43.2, 115.2 (2C), 123.0 (2C), 125.6, 128.8 (2C), 129.0 (2C), 138.3, 138.8 (2C), 140.7, 141.5, 147.2, 163.2. MS (*m*/*z*, %): 348 (M^+^, 1.06). HRMS calc. forC_18_H_16_N_6_S: 348.1157, found: 348.1168.

*1-(4-((2-(((1H-benzimidazol-2-yl)methyl)amino)thiazol-5-yl)diazenyl)phenyl)ethan-1-one* (**3c**).Brown solid, M.p.: >250 °C, Yield: 1.7 g, 47%. IR: *ν*_max_ = 3740–2850 (NH, =CH aromatic, –CH aliphatic), 1667 (CO acetyl), 1619 (C=N), 1453 (CH_2_), and 1388 (CH_3_) cm^−1^. ^1^H-NMR: *δ*/ppm = 2.44 (s, 2H, CH_2_ aliphatic), 3.43 (s, 3H, CH_3_), 5.87 (s, 1H, NH), 7.16–7.74 (m, 8H, Ar–H; s, 1H, C_4_–thiazole–H), 10.90 (s, 1H, NH-benzimidazole ring). ^13^C-NMR: *δ*/ppm = 26.3, 43.3, 115.2 (2C), 123.0 (2C), 128.8 (4C), 133.1, 136.8, 138.8 (2C), 140.7, 141.5, 147.3, 163.2, 197.0. MS (*m*/*z*, %): 376 (M^+^, 100.0). HRMS calc. for C_19_H_16_N_6_OS: 376.1106, found: 376.1118.

#### 3.4.4. Synthesis of 1-((1*H*-Benzimidazol-2-yl)Methyl)Thiourea (**4**)

A mixture of 2-(bromomethyl)-1*H*-benzimidazole **1** (2.11 g, 10 mmol) and thiourea (1.14 g, 15 mmol) in ethanol (30 mL) containing catalytic amount of trifluoroacetic acid (0.02 mL), was well stirred under reflux for 2 h. The reaction mixture was allowed to cool, and the solid product that was formed was filtered off, washed with cold ethanol, dried, and recrystallized from ethanol to obtain a brown solid; M.p.: 129–130 °C, Yield: 2 g, 99%. IR: *ν*_max_ = 3740 (NH_2_), 3251–3020 (NH, =CH aromatic, –CH aliphatic), 1601 (C=N) and 1485(CH_2_) cm^−1^. ^1^H-NMR: *δ*/ppm = 3.39 (d, 2H, *J* = 7.4 Hz, CH_2_ aliphatic), 5.87 (t, 1H, NH), 7.18–7.48 (m, 4H, Ar–H), 9.50 (s, 2H, NH_2_), 10.90 (s, 1H, NH–hydrazo), 11.31 (s, 1H, NH–benzimidazole ring). ^13^C-NMR: *δ*/ppm = 44.7, 115.2 (2C), 123.0 (2C), 138.8 (2C), 141.5, 182.5. MS (*m*/*z*, %): 206 (M^+^, 100.0). HRMS calc. for C_9_H_10_N_4_S: 206.0626, found: 206.0649 [45].

#### 3.4.5. Synthesis of (2-((1*H*-Benzimidazol-2-yl)Methyl)Amino)Thiazo-4(5*H*)-One (**5**)

To a well stirred solution of 1-((1*H*-benzimidazol-2-yl)methyl)thiourea **4** (2.06 g, 10 mmol) in 20 mL of ethanol containing 0.5 mL of triethylamine (TEA), a chloroacetylchloride (1.12 mL, 10 mmol) was added over one h at room temperature. The reaction mixture was then heated, and stirring was continued at 60 °C for five h. The mixture was allowed to cool, and the solid product formed was collected, dried, and recrystallized from ethanol to yield orange crystals; M.p.: >250 °C (284 °C [59]), Yield: 1.8 g, 75%.

#### 3.4.6. Synthesis of *N*-((1*H*-Benzimidazol-2-yl)Methyl)-4-Phenylthiazol-2-Amine (**6**)

A mixture of 1-((1*H*-benzimidazol-2-yl)methyl)thiourea **4** (2.06 g, 10 mmol) in 20 mL of ethanol containing a few drops of piperidine, and phenacylchloride (1.54 g, 10 mmol) was allowed to stir under reflux for six h. The red dish brown solid product formed was collected, washed from methanol, dried, and recrystallized from ethanol; M.p.: 219–220 °C, Yield: 2.7 g, 88%. IR: *ν*_max_ = 3366–2950 (NH, =CH aromatic, –CH aliphatic), 1629 (C=N), and 1444(CH_2_) cm^−1^. ^1^H-NMR: *δ*/ppm = 2.49 (s, 2H, CH_2_ aliphatic), 5.32 (s, 1H, NH), 7.30–7.64 (m, 9H, Ar–H), 7.90 (s, 1H, C–4 thiazole ring), 10.10 (s, 1H, NH–benzimidazole ring). ^13^C-NMR: *δ*/ppm = 43.3, 107.8, 115.2 (2C), 123.0 (2C), 127.5, 128.4 (2C), 134.8 (2C), 136.3, 138.8 (2C), 141.5, 158.2, 161.3. MS (*m*/*z*, %): 306 (M^+^, 18.4). HRMS calc. for C_17_H_14_N_4_S: 306.0939, found: 306.0960.

#### 3.4.7. General Procedure for the Synthesis of the Arylazo Derivatives (**7a**–**c**) and (**8a**–**c**)

The corresponding aryl diazonium chlorideswere prepared by adding cold sodium nitrite solution 0.69 g, 10 mmol in 15 mL of H_2_O) to a cold suspension of different aromatic amines (10 mmol) in concentrated HCl (4–6 mL) with continuous stirring. To a cold solution of (2-((1*H*-benzimidazol-2-yl)methyl)amino)thiazo-4(5*H*)-one **5** (2.46 g,10 mmol) and/or *N*-((1*H*-benzimidazol-2-yl)methyl)-4-phenylthiazol-2-amine**6**(3.06 g, 10 mmol) in ethanol (20 mL) and sodium acetate (1.6 g, 20 mmol), a cold aqueous solution from the corresponding aromatic diazonium salts were added dropwise with stirring at 0–5 °C for two h. The solid products obtained were filtered off, washed with water followed by cold ethanol, dried, and recrystallized from ethanol to give 2-(((1*H*-benzimidazol-2-yl)methyl)imino)-5-(2-aryldiazenyl)thiazolidin-4-one (**7a**–**c**) and *N*-((1*H*-benzimidazol-2-yl)methyl)-5-(aryldiazenyl)-4-phenylthiazol-2(3*H*)-imine (**8a**–**c**).

*2-(((1H-benzimidazol-2-yl)methyl)amino)-5-((4-nitrophenyl)diazenyl)thiazol-4(5H)-one* (**7a**). Dark brown solid; M.p.: >250 °C, Yield: 2.9 g, 73%. IR: *ν*_max_ = 3366–2961 (NH, =CH aromatic, –CH aliphatic), 1635 (CO), 1585 (C=N), 1438 (CH_2_) and 1332, 1519 (NO_2_) cm^−1^. ^1^H-NMR: *δ*/ppm = 2.50 (s, 2H, CH_2_ aliphatic), 4.11 (s, 1H, NH), 7.18–7.89 (m, 8H, Ar–H), 9.70 (s, 1H, NH–benzimidazole ring). ^13^C-NMR: *δ*/ppm = 43.5, 74.6, 115.2 (2C), 120.5 (2C), 123.0 (2C), 124.1 (2C), 138.8 (2C), 141.5, 157.4, 157.0, 158.2, 173.3. MS (*m*/*z*, %): 395 (M^+^, 0.45). HRMS calc. for C_17_H_13_N_7_O_3_S: 395.0801, found: 395.0822.

*2-(((1H-benzimidazol-2-yl)methyl)amino)-5-(p-tolyldiazenyl)thiazol-4(5H)-one* (**7b**). Dark brown solid; M.p.: >250 °C, Yield: 1.9 g, 53%. IR: *ν*_max_ = 3627–2799 (NH, =CH aromatic, –CH aliphatic), 1654 (CO), 1595 (C=N), 1441 (CH_2_) and1408 (CH_3_) cm^−1^. ^1^H-NMR: *δ*/ppm = 2.50 (s, 3H, CH_3_), 3.08(s, 2H, CH_2_ aliphatic), 4.89 (s, 1H, NH), 7.21–8.48 (m, 8H, Ar–H) and 8.91 (s, 1H, NH–benzimidazole ring). ^13^C-NMR: *δ*/ppm = 21.3, 43.7, 74.6, 115.2 (2C), 122.5 (2C), 123.0 (2C), 129.1 (2C), 138.8 (2C), 141.5, 147.7, 157.7, 158.2, 173.4. MS (*m*/*z*, %): 364 (M^+^, 0.18). HRMS calc. for C_18_H_168_N_6_OS: 364.1106, found: 364.1131.

*2-(((1H-benzimidazol-2-yl)methyl)amino)-5-((4-acetylphenyl)diazenyl)thiazol-4(5H)-one* (**7c**). Brown solid; M.p.: >250 °C, Yield: 1.9 g, 49%. IR: *ν*_max_ = 3627–2990 (NH, =CH aromatic, –CH aliphatic), 1660 (CO), 1591 (C=N), 1431(CH_2_) and 1402 (CH_3_) cm^−1^. ^1^H-NMR: *δ*/ppm = 2.49 (s, 3H, CH_3_), 3.32 (s, 2H, CH_2_ aliphatic), 4.11 (s, 1H, NH), 7.16–7.98 (m, 8H, Ar–H) and 9.54 (s, 1H, NH–benzimidazole ring). ^13^C-NMR: *δ*/ppm = 26.4, 43.7, 74.6, 115.2 (2C), 122.5 (2C), 123.0 (2C), 129.1 (2C), 133.6, 138.8 (2C), 141.5, 155.1, 158.2, 173.3, 197.0. MS (*m*/*z*, %): 393.11 (M^+^, 20.5). HRMS calc. for C_19_H_15_N_6_O_2_S: 392.1055, found: 392.1071.

*N-((1H-benzimidazol-2-yl)methyl)-5-((4-nitrophenyl)diazenyl)-4-phenylthiazol-2-amine* (**8a**). Brown solid; M.p.: 183–185 °C, Yield: 3.2 g, 72%. IR: *ν*_max_ = 3366–2950 (NH, =CH aromatic, –CH aliphatic), 1629 (C=N), 1444 (CH_2_) and 1322, 1513 (NO_2_) cm^−1^. ^1^H-NMR: *δ*/ppm = 2.50 (s, 2H, CH_2_ aliphatic), 5.32 (s, 1H, NH), 7.52–8.31(m, 13H, Ar–H), 8.99 (s, 1H, NH–benzimidazole ring). ^13^C-NMR: *δ*/ppm = 44.2, 89.6, 115.2 (2C), 123.0 (2C), 123.8 (2C), 126.5 (2C), 127.9, 128.3 (2C), 128.6 (2C), 134.1, 138.8 (2C), 141.5, 147.8, 154.1, 158.2, 161.0. MS (*m*/*z*, %): 455 (M^+^, 24.9). HRMS calc. for C_23_H_17_N_7_O_2_S: 455.1164, found: 455.1145.

*N-((1H-benzimidazol-2-yl)methyl)-4-phenyl-5-(p-tolyldiazenyl)thiazol-2-amine* (**8b**). Brown solid; M.p.: 162–163 °C, Yield 4 g, 95%. IR: *ν*_max_ = 3385–2964 (NH, =CH aromatic, –CH aliphatic), 1636 (C=N),1475 (CH_2_), and 1407 (CH_3_) cm^−1^.^1^H-NMR: *δ*/ppm = 2.25 (s, 3H, CH_3_), 3.44 (s, 2H, CH_2_ aliphatic), 4.22 (s, 1H, NH), 7.09–7.57 (m, 13H, Ar–H), 10.14 (s, 1H, NH–benzimidazole ring). ^13^C-NMR: *δ*/ppm = 21.3, 44.2, 89.6, 115.2 (2C), 123.0 (2C), 127.8, 128.3 (2C), 128.6 (2C), 128.7 (2C), 129.0 (2C), 134.1, 138.4, 138.8 (2C), 141.5, 145.1, 158.2, 161.0. MS (*m*/*z*, %): 424 (M^+^, 1.36). HRMS calc. for C_24_H_20_N_6_S: 424.1470, found: 424.1452.

*1-(4-((2-(((1H-benzimidazol-2-yl)methyl)amino)-4-phenylthiazol-5-yl)diazenyl)phenyl)ethan-1-one* (**8c**).Brown solid; M.p.: 130–131 °C, Yield: 4.3 g, 96%. IR: *ν*_max_ = 3295–2855 (NH, =CH aromatic, –CH aliphatic), 1656 (CO acetyl), 1588 (C=N), 1474 (CH_2_), and 1407 (CH_3_) cm^−1^. ^1^H-NMR: *δ*/ppm = 2.54 (s, 3H, CH_3_), 3.43 (s, 2H, CH_2_ aliphatic), 4.32 (s, 1H, NH), 7.31–8.43 (m, 13H, Ar–H) and 10.94 (s, 1H, NH–benzimidazole ring). ^13^C-NMR: *δ*/ppm = 26.4, 44.2, 89.6, 115.2 (2C), 123.0 (2C), 128.3 (2C), 128.6 (5C), 128.7 (2C), 134.1, 136.4, 138.8 (2C), 141.5, 152.2, 158.2, 161.0, 197.0. MS (*m*/*z*, %): 452 (M^+^, 0.23). HRMS calc. for C_25_H_20_N_6_OS: 452.1419, found: 452.1423.

## 4. Conclusions

In the development of this work, the newly synthesized series of benzimidazole derivatives **3a**–**c**, **7a**–**c**, and **8a**–**c** derived from 2-(bromomethyl)-1*H*-benzimidazole were prepared in satisfactory yields and fully characterized. They exhibited in vitro variable antimicrobial activities when subjected to the bacterial strains *Staphylococcus aureus* (Gram-positive bacteria) and *Escherichia coli* (Gram-negative bacteria) compared with *Kanamycin* and *Ampicillin* as standard drugs, respectively. Their inhibition effect against DPPH (the lowest EC_50_ values) compared to Trolox (EC_50_ = 55.13) was shown as well. They were screened for their antitumor activities against human liver cancer cell line (HepG2) to evaluate the cytotoxic effects of gradient concentrations (100–500 μg/mL) in terms of cell viability percentage and EC_50_ values. The tested compounds exhibited noticeable efficiency against the tumor cell line under investigation at the higher concentration. The benzimidazole derivatives (**3a**, **c**) and (**8b**) showed higher activity than the other selected compounds that were tested. Presence of electron withdrawing groups increased the antitumor activities as discussed.

## Figures and Tables

**Table 1:** The minimum inhibitory concentration (MIC) of effective tested compounds and positive reference standards.

			MIC, µg/mL (Average ± SD)								
Tested Strains	Reference Control				Tested Compounds						
	**Ampicillin**	**Kanamycin**	**3a**	**3b**	**3c**	**7a**	**7b**	**7c**	**8a**	**8b**	**8c**
*E. coli*	2.1 ± 0.09	-	-	-	14 ± 1.54	-	-	-	16 ± 1.05	-	12 ± 0.095
*S. aureus*	-	11.3 ±0.87	32 ± 3.95	24 ± 2.21	-	-	-	27 ± 1.23	28 ± 1.3	-	21 ± 1.64

*E. coli*: Escherichia coli; *S. aureus*: Staphylococcus aureus, Values are mean ± SD, *n* = 3.
